# The effectiveness of ultrasonography in verifying the placement of a nasogastric tube in patients with low consciousness at an emergency center

**DOI:** 10.1186/1757-7241-20-38

**Published:** 2012-06-12

**Authors:** Hyung Min Kim, Byung Hak So, Won Jung Jeong, Se Min Choi, Kyu Nam Park

**Affiliations:** 1 Department of Emergency Medicine, College of Medicine, The Catholic University of Korea, St. Vincent’s Hospital, Suwon, South Korea; 2 Department of Emergency Medicine, College of Medicine, The Catholic University of Korea, St. Mary’s Hospital, Uijeongbu, South Korea; 3 Department of Emergency Medicine, College of Medicine, The Catholic University of Korea, St. Mary’s Hospital, Seoul, South Korea

**Keywords:** Nasogastric tube, pH, Ultrasonography, Auscultation

## Abstract

**Background:**

This study was designed to compare the effectiveness of using auscultation, pH measurements of gastric aspirates, and ultrasonography as physical examination methods to verify nasogastric tube(NGT) placement in emergency room patients with low consciousness who require NGT insertion.

**Methods:**

The study included 47 patients who were all over 18 years of age. In all patients, tube placement was verified by chest X-rays. Auscultation, pH analysis of gastric aspirates, and ultrasonography were conducted on each patient in random order. The mean patient age was 57.62 ± 17.24 years, and 28 males (59.6%) and 19 females (40.4%) were included. The NGT was inserted by an emergency room resident. For pH testing, gastric aspirates were dropped onto litmus paper, and the resulting color of the paper was compared with a reference table. Ultrasonography was performed by an emergency medicine specialist, and the chest X-ray examination was interpreted by a different emergency medicine specialist who did not conduct the ultrasonography test. The results of the auscultation, gastric aspirate pH, and ultrasonography examinations were compared with the results of the chest x-ray examination.

**Results:**

The sensitivity and specificity were 100% and 33.3%, respectively, for auscultation and 86.4% and 66.7%, respectively, for ultrasonography. Kappa values were the highest for auscultation at 0.484 compared to chest x-rays, followed by 0.299 for ultrasonography and 0.444 for pH analysis of the gastric aspirate. The ultrasonography has a positive predictive value of 97.4% and a negative predictive value of 25%.

**Conclusions:**

Ultrasonography is useful for confirming the results of auscultation after NGT insertion among patients with low consciousness at an emergency center. When ultrasound findings suggest that the NGT placement is not gastric, additional chest X-ray should be performed.

## Introduction

NGT insertion is one of the most commonly performed procedure in an emergency setting. It is usually performed in patients who are being treated for intestinal adhesions, suspicion of gastric bleeding, overdose, or who require mechanical ventilation after endotracheal intubation. Whilst the misplacement rate appears low, the complications may be very serious. A major complication of this procedure is the aspiration of gastric contents which could lead even to death, especially high in patients with low consciousness and increases if food or medicine is administered through a NGT whose port is incorrectly placed in the mouth, the esophagus, or the esophagogastric junction [1-4]. Aspiration can be caused by regurgitation during the insertion of the tube, esophageal perforation, or accidental placement of the tube in the respiratory tract or the cranial cavity. Even when the tube is correctly placed within the stomach, it can be dislocated when the patient coughs, sneezes, or vomits.

Several methods have been suggested for verifying the placement of a NGT, including auscultation, measuring the pH of aspirates from the tube, and chest x-rays. In addition, the use of colorimetric capnography has been demonstrated recently [5,6]. Auscultation with a stethoscope confirms gurgling sounds in the epigastrium when air is injected after NGT insertion. However, in the noisy environment of an emergency room, sounds associated with the incorrect placement of the NGT in the lungs or in the esophagus might be mistaken for those associated with the correct placement of the tube, and a basic chest X-ray is recommended in most cases [7-9]. But chest X-rays have issues of their own, including delayed verification, radiation exposure, and cost. In most emergency rooms, chest X-rays are not performed immediately after NGT insertion. Because the verification of tube placement instead usually relies on auscultation, a significant risk of complications remains, especially in patients with low consciousness. Aspirating the nasogastric tube contents and using litmus paper to measure the pH of the aspirates is an alternative method for verifying tube placement. This method has been reported to be effective for verifying the placement of the nasogastric feeding tube and for the continuous monitoring of intensive care unit patients [10-12]. However, to date, no other recommendations or alternative methods have been proposed for emergency room settings. In addition, ultrasonography is currently used in many settings, including the emergency room. The frequent use of this procedure according to the needs of the patient is helpful for making the correct medical judgments. Therefore, this study was designed to compare the effectiveness and the limitations of conventional auscultation, pH analysis of the tube aspirate, chest X-ray, and ultrasonography for verifying the placement of a NGT in patients with low consciousness at an emergency center.

## Methods

### Settings and patients

This prospective study was conducted over 5 months (from May to September 2011) at a local emergency center visited by 55,000 patients annually. Participants in the trial included patients with low consciousness in whom correct placement of the NGT was ultimately verified by chest X-ray. All patients were over 18 years of age and underwent NGT insertions for reasons including drug overdose, suspicion of gastric bleeding, endotracheal intubation, and others.

### Outcome measures

NGT insertion was performed bedside by a emergency room resident physician by measuring the distance from the tip of the patient’s nose to the earlobe and from the earlobe to the xiphisternum. NGTs were inserted to a length 10 cm longer than the distance obtained by this measurement. The tube size was 16 Fr. After the insertion, auscultation, pH testing of the tube aspirate, and ultrasonography (GE LOGIQ 400, USA) were conducted in random order, and the results were recorded.

Auscultation was performed by assessing sounds in the epigastrium while injecting 10–20 cc of air into the NGT with a 50 cc syringe. The results of this examination were also recorded. The gastric pH analysis was performed by dropping a sample that was aspirated from the tube onto a litmus strip (TOYO ROHIO CO. LTD). The pH reading was recorded based on color-coded reference values, and the tube placement was considered to be gastric if the pH was less than 5 [10]. Ultrasound examinations were conducted by 2 emergency medicine specialists who received basic training on the routine use of ultrasound to verify NGT insertion. Final confirmation of the gastric placement of the tube was obtained by chest X-ray that is the test method reference standard to confirm correct NGT placement. Chest X-rays were interpreted by an emergency medicine specialist who did not perform the ultrasound examinations.

**Figure 1:**
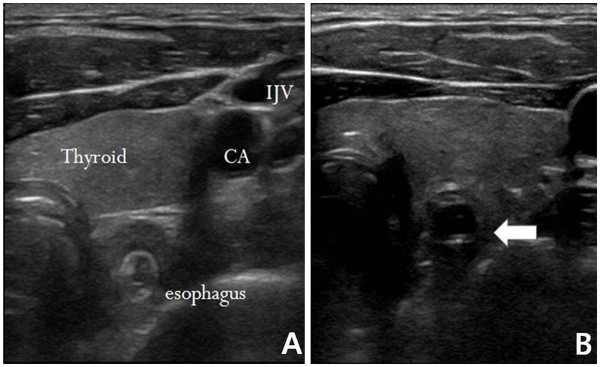
**Normal esophagus (A).** White arrow indicates the nasogastric tube in the esophagus (**B**).

**Figure 2:**
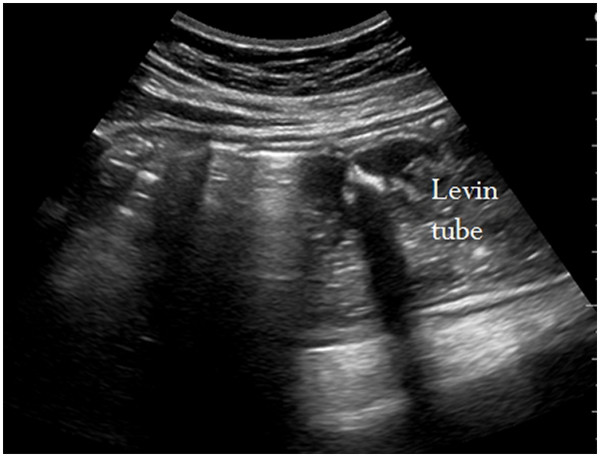
**Sonographic visualization of a nasogastric tube.** The weighted tip appears as a hyperechogenic line with a posterior acoustic shadow.

**Figure 3:**
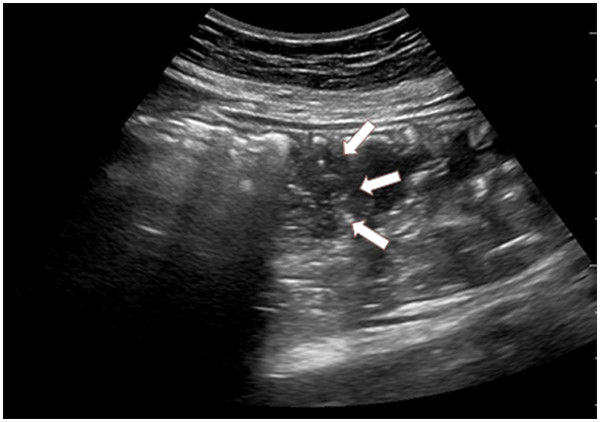
A mixture of 40 ml normal saline with 10 ml air was injected into the tube to visualize the hyperechogenic "fog" existing at the tip of the tube (white arrows).

Ultrasound examinations included a transversal scan that was performed prior to tube insertion from either the right or left side of the patient’s neck. This scan was performed to verify that the esophagus was located behind the respiratory tract. If attenuated ultrasound waves in the far field and the posterior wall of the esophagus were not observed after tube insertion, the NGT was considered to be positioned within the cervical esophagus (Figure 1). In the esophagogastric junction, the NGT was directly visualized with longitudinal and angled scans of the epigastrium. Visualization of the NGT in separate scans of the fundus and the antrum of stomach was attempted. We used linear probe for the study of the neck and convex probe for stomach. If visualization was not possible, 40 cc of normal saline and 10 cc of air were injected through the NGT and if ultrasonography showed dynamic fogging in the stomach, gastric placement of the tube was verified (Figures 2 and 3). This study was approved by the Bioethics Committee at the Catholic University of Korea.

### Statistical methods

In the descriptive statistical analysis, continuous variables were reported in terms of the mean and standard deviation, and nominal variables were reported in terms of frequency and percentage. Sensitivity, specificity, positive predictive value, and negative predictive value were calculated to assess the diagnostic ability of the techniques studied in this research, and Cohen’s kappa analysis was conducted to evaluate the degree of concordance. SPSS for Windows (ver. 18.0, SPSS Inc., Chicago, USA) was used for statistical analysis. P values less than 0.05 were considered to indicate statistical significance.

## Results

A total of 74 patients were screened for the study. Seventeen patients with normal levels of consciousness were excluded. Ten more patients were excluded because they did not undergo a radiological examination after tube insertion. Thus, a total of 47 patients composed the study sample. The mean age of the patients was 57.62 ± 17.24 years. Among the patients with a Glasgow Coma Scale (GCS) score lower than 14, 27 patients (57.4%) underwent endotracheal intubation because of pneumonia, overdose, external injury, or CPR. This group accounted for the largest share of the patients, followed by poisoning, gastrointestinal bleeding, and intestinal adhesions (Table 1).

**Table 1:** Characteristics of patients

**Characteristics**	**n = 47**
Age (years)	57.6 ± 17.2^*^
Gender (male/female)	28(59.6)/19(40.4)^†^
Nasogastric tube indications	
Poisoning	12(25.5)
Ileus	2(4.3)
Intubation	27(57.4)
Gastrointestinal bleeding	6(12.8)
GCS**	
≤8	33(70.2)
9-14	14(29.8)

^*^Mean ± SD, SD: standard deviation.

^†^Number (%).

**Glasgow coma scale.

Of the 47 patients who underwent chest X-rays, the NGT was verified to be in the stomach for 44 patients (93.6%). In the remaining 3 patients, the tube had been placed in the esophagus. None of the tubes had been placed in the bronchial tubes or in the cranial cavity. Among the methods for tube placement verification tested in this study, auscultation had the highest sensitivity at 100%; however, its specificity was low at 33.3. Auscultation suggested that the tube was in the stomach when it was in fact in the esophagus in 2 patients (1 patient with intestinal adhesion and 1 who had undergone endotracheal intubation).

Of the 3 patients with incorrectly diagnosed gastric placement of the NGT based on pH analysis, the pH was greater than 5, and 2 of these patients had received anti-ulcer medication through a NGT, and 1 patient was alkali poisoned. The gastric contents were not aspirated in 10(21.3%) patients(6 patients who received endotracheal intubation after bag-valve-mask ventilation, 1 patient with overdose, and 3 patients with gastrointestinal bleeding)(Table 2).

**Table 2:** Comparison of predictive validity

**Evaluation method**	**Total (n = 47)**		**Sensitivity (%)**	**Specificity (%)**	**PPV (%)**	**NPV (%)**
	**correct position (n = 44)**	**incorrect position (n = 3)**				
Ultrasound (+)	38	1	86.4	66.7	97.4	25.0
(−)	6	2				
Auscultation (+)	44	2	100.0	33.3	95.7	100.0
(−)	0	1				
pH (+)	31	1	91.2	66.7	96.9	40
measuring^*^ (−)	3	2				

PPV: positive predictive value.

NPV: negative predictive value.

^*^ pH measuring: The contents were not aspirated in 10 patients.

Ultrasound had a lower sensitivity than auscultation (86.4%) but had a higher specificity (66.7%) and positive predictive value(97.4%). Ultrasonography failed to verify gastric placement of the tube in 6 patients (3 patients who had undergone endotracheal intubation, 2 with overdose, and 1 with gastrointestinal bleeding). In another patient, the tube had been placed in the esophagus, but fogging led to a diagnosis of gastric placement.

The kappa value for each test was highest for auscultation at 0.484 compared with chest X-ray examinations, and the kappa value was 0.299 for ultrasonography and 0.444 for pH analysis(Table 3).

**Table 3:** Number of correct classification and results of Cohen Kappa analysis

**Evaluation method**	**Correct placement**	**Incorrect placement**	**K**	**p**
Radiology	44^*^(93.6)	3 (6.4)		
Ultrasound	39 (83.0)	8 (17.0)	0.299	0.018
Auscultation	46 (97.9)	1 (2.1)	0.484	<0.001
pH measuring^†^	32 (86.5)	5 (13.5)	0.444	0.005

^*^Number (%).

^†^ pH measuring: The contents were not aspirated in 10 patients.

Ultrasonography visualized the NGT in the neck area in 39 (83%) patients. In the gastro-esophageal junction, visualization of the nasogastric feeding tube was impaired due to artifacts produced by air in 8 patients (17%). The tube was directly visualized in the stomach in 6 patients (12.8%), and fogging occurred in the remaining 33 patients; these results were due to gas interposition(Table 4).

**Table 4:** The results of ultrasonography

**Site**	**Visualization of tube**	**Fogging**
Neck	39^*^(83.0)	
Gastric-esophageal		
junction	8 (17.0)	
Stomach	6 (12.8)	33 (70.2)

^*^Number (%).

## Discussion

Auscultation could be mistaken for those associated with the correct placement of the NGT because bronchic insertion can lead to gurgling perceived in the epigastric area. Metheny *et al.* have reported that auscultation has an accuracy of 34.4% for verifying the placement of a NGT [8]. In an emergency room setting, which is often noisy, an auxiliary method of verifying tube placement is necessary.

In a study by Turgay *et al.* of 44 patients in intensive care, a nurse inserted the NGT and verified the placement of the tube by auscultation and pH measurement. Auscultation diagnosed gastric placement in 90.9% of the patients, whereas radiographic examination showed that the tube was in the stomach in only 88.6% of patients. However, pH measurements agreed with radiological examinations in 94.9% of cases. Thus, Turgay *et al.* argued that pH measurement was a more accurate and reliable test than auscultation. It has also been reported that bedside pH measurements can drastically reduce the necessity of radiological examination [10]. But, we could not determine the tube placement in 10 patients (21.3%) because the contents were not aspirated. In emergency room patients with low consciousness, aspiration of the gastric contents (even in patients with a nasogastric tube in place) was frequently impossible due to either bag-valve-mask ventilation during intubation or the presence of large amounts of gastric air in patients with intestinal obstruction or paralytic intestinal adhesion. Also, Some study also reported that the pH method was inappropriate for distinguishing tube placement within the bronchial tubes or the small intestine in patients with reduced gastric acidity [13,14]. In this study, the complete history of the patients’ use of medications, such as H2 blockers, was not known. However, pH analysis provided false negative results in several cases, including in a patient who was taking a stomach medication and was admitted to the emergency room due to gastrointestinal bleeding, in a patient who was routinely administered food and medication through a NGT, and in a patient with alkali poisoning. These findings suggest that verifying NGT placement with the pH method alone has limitations, and interpretation of these data should be made with careful consideration of the clinical status and medical history of each patient.

Ultrasonography is convenient, fast, noninvasive, and has been widely used as a diagnostic examination technique since its introduction. It has almost no spatial or temporal restrictions, and its effectiveness as a diagnostic tool and procedure has been established worldwide. Vigneau *et al.* conducted a double-blind experiment in which a fellow who received brief (approximately 2 hours) training successfully inserted the NGT into 33 intensive care unit patients. This study showed that ultrasonography had 97% sensitivity and could be performed in a shorter time than conventional radiological examinations. The authors also suggested that a basic chest x-ray should be taken in patients in whom tube placement could not be verified using ultrasonography [15]. A study by Chenaitia H. *et al.* also found that NGT placement could be easily determined by ultrasonography in prehospital managment [14]. This shows that ultrasonography is a possible method for verifying the placement of the NGT.

The present study yielded a lower accuracy for ultrasonography than other studies for several reasons. First, because the patients had a low consciousness level in this study, the examination was performed with the patients in a supine position. In cases of obese or excessively mobile patients, it was difficult to posteriorly observe the NGT in the cervical region. Furthermore, it was often difficult to visualize the esophagogastric junction or the NGT directly because of the large volume of gas in the gastrointestinal tract. However, even without direct visualization, gastric placement of the NGT could be verified by ultrasound in 33 patients (70.2%) through the fogging that occurred with the injection of normal saline. In this study, the high positive predictive value of ultrasonography could surely reduced the number of chest X-ray and the visualization of the tube directly by ultrasonography or after water and air insufflations rules out incorrect placement. However obviously still requires someone skilled to interpret the images and training to perform the testing.

One limitation of this study includes the difficulty of directly analyzing the accuracy of the ultrasonography examinations due to the low number of cases of incorrect insertions of the NGT. For more accurate results, it will be necessary to conduct studies on a larger number of patients in the future.

## Conclusions

When inserting the NGT into a patient with low consciousness at an emergency center, placement can be first verified by auscultation. Because pH analysis of the tube aspirate is not possible in some patients and is subject to false negative results in others, we do not recommend that this method be used for secondary verification of tube placement. Verifying NGT placement with ultrasonography has the potential to reduce complications, save time, and reduce unnecessary radiation exposure. However, for cases in which ultrasound cannot verify placement of the NGT by direct visualization or after water and air insufflation, confirmation with chest x-ray is necessary.

## Competing interests

The authors declare that they have no competing interests.

## Authors' contributions

HMK drafted the manuscript. BHS, WJJ, SMC and KNP reviewed data and revised the manuscript. WJJ and SMC managed the data and reviewed critical revisions to the manuscript. BHS and KNP performed data analysis and revised the manuscript. HMK conceived the research and drafted the manuscript. All authors have read and approved the final manuscript.
